# REVISITING POSITIVE AFFECT: THE ROLE OF AFFECT MEAN LEVELS AND VARIABILITY ON DEPRESSION IN MID AND LATER LIFE

**DOI:** 10.1093/geroni/igad104.0927

**Published:** 2023-12-21

**Authors:** Sun Ah Lee, Dahlia Mukherjee, David Almeida

**Affiliations:** The Pennsylvania State University, University Park, Pennsylvania, United States; Penn State College of Medicine, Hershey, Pennsylvania, United States; The Pennsylvania State University, State College, Pennsylvania, United States

## Abstract

Recent research suggests that affect dynamics (e.g., variability) combined with mean levels are important predictors of psychological and physical health outcomes. This study explores the joint effect of affect levels and variability on depression across 10 years among middle-aged and older adults. We used the data from the second and third waves of Midlife in the United States (MIDUS) study. During telephone interviews, daily positive (PA) and negative affect (NA) were assessed across eight consecutive evenings. Daily affect mean levels were calculated by averaging daily affect across interview days, and daily affect variability was calculated as the individual’s standard deviation (iSD). Depression status was assessed using the Composite International Diagnostic Interview-Short form at baseline and 10-year follow-up. The final analytic sample includes 1,499 adults aged 55 years old on average (age range = 34-83; 55.7% female; 93% white). Logistic regression analysis was performed to test the interaction effect of daily affect mean levels and variability on depression. We found a significant interaction effect between PA mean levels and variability on depression status at follow-up. For individuals with high PA levels, higher PA variability was associated with higher odds of developing depression, after adjusting for depression and anxiety disorder at baseline. For NA, we only found main effects where higher NA variability was associated with higher odds of depression at follow-up. Findings suggest fragile high PA (i.e., variable high PA) may be a significant predictor of depression, highlighting the need for understanding PA as multidimensional construct.

